# Biocompatibility of Amalgam vs Composite – A Review

**DOI:** 10.3290/j.ohpd.b2831749

**Published:** 2022-03-21

**Authors:** Gottfried Schmalz, Matthias Widbiller

**Affiliations:** a Professor, Department of Conservative Dentistry and Periodontology, University Hospital Regensburg, Germany; Department of Preventive, Restorative and Pediatric Dentistry, University of Bern, Bern, Switzerland. Project elaboration, conceptual ideas, wrote the manuscript.; b Assistant Professor, Department of Conservative Dentistry and Periodontology, University Hospital Regensburg, Germany. Project elaboration, conceptual ideas, wrote the manuscript.

**Keywords:** bisphenol-A, child, lactation, mercury, pregnancy

## Abstract

**Summary:**The Minamata Convention resulted in restrictions in the use of amalgam in daily dental practice. This opens up new discussions about the biocompatibility of amalgam, but also of composites as alternative materials. In the following review article, these issues will be discussed in more detail to provide dentists with a knowledge base for themselves and for communication with their patients. In addition to mercury in amalgam or monomers in composites, bisphenol A and nanoparticles generated during the grinding, polishing or removal of restorations must also be included in the biocompatibility evaluation. In laboratory tests, these substances cause toxic reactions, and bisphenol A also exhibits estrogen-like effects. However, it must be taken into account that the concentrations used in laboratory tests are much higher than in clinical practice. Thus, both amalgam and composite can be used in the general population. Nevertheless, for scientifically, politically and legally defined risk groups (e.g. dental personnel, allergic persons, pregnant or lactating women, children under 15 years of age, people with certain systemic diseases), indication restrictions and precautionary measures must be observed. The well-known amalgam discussion has taught us the importance of thorough and open risk communication with the patient.

The Minamata Convention on the reduction of mercury emissions into the environment and its implementation in European law are currently giving rise to renewed discussion about the biocompatibility not only of amalgam, but also of composite, which is widely used worldwide as an alternative restorative material to amalgam.^12,17^ The impact of the new regulation of the European Union (EU) on the use of amalgam in daily practice has already been extensively discussed in the literature.^16,19^ With regard to the increasing use of composites, dentists are confronted with the provocative question of whether the cure is worse than the disease. Moreover, the unmistakable parallels in the debate on the biocompatibility of amalgam and composites suggest that experience gained with amalgam should be drawn upon: How should dentists react sensibly and argue effectively with regard to the discussion about the biocompatibility of composites, avoiding both inaccurate trivialisation and undue outrage?

## Tissue Exposure

**Fig 1:**
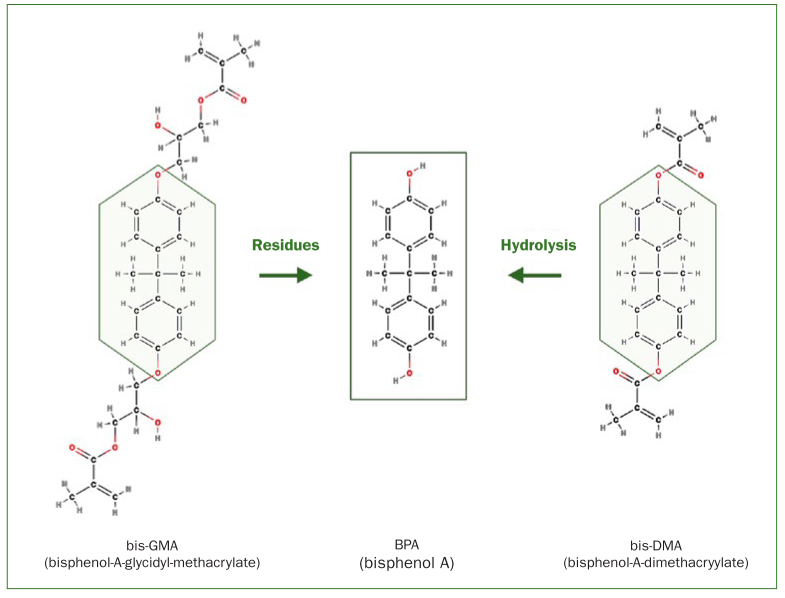
Bisphenol A is used in the manufacture of bis-GMA, and residues of it can leach from dental materials into the oral cavity in small amounts. Hydrolytic degradation of bis-DMA, which may be present in resins used for fissure sealing, also leads to the release of bisphenol A under certain circumstances.

**Fig 2:**
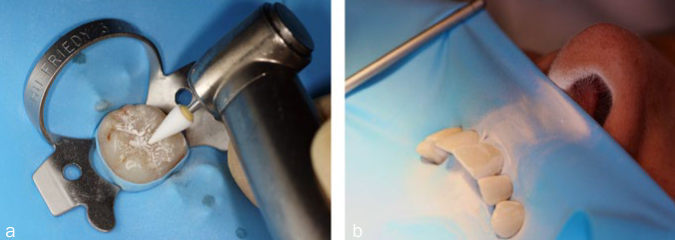
Generation of dusts and nanoparticles. (a) Dry processing and polishing of composite fillings generate grinding dust containing nanoparticles. However, prolonged dry processing is not recommended in order to keep the amount of dust low. (b) The resulting dust and nanoparticles can be inhaled by the patients as well as the treatment team (reprinted with kind permission of Stevan M. Cokic, KU Leuven/Belgium).

The biological effect of dental materials only unfolds during the exposure of living tissue through the release of certain substances. For example, very small amounts of mercury are released from amalgam.^13^ Composites also release many different substances, such as monomers (e.g. bis-GMA, UDMA, TEG-DMA, HEMA), catalysts, accelerators or other residues such as bisphenol A.^13^ In areas outside dentistry, the release of bisphenol A (Fig 1) also occurs from polycarbonate and epoxy-based plastics (beverage bottles, disposable cutlery, etc). Epoxy resins are also used for the internal coating of beverage and food cans. Furthermore, bisphenol A is required for the production of bis-GMA, the most common base molecule of composites, and can be detected in it in small quantities as an impurity or residue.^14^ Bis-GMA itself does not degrade to bisphenol A under physiological conditions in the oral cavity.^2,25^ In contrast, bisphenol A can split off in the saliva environment from bis-DMA, which is contained in some materials for fissure sealing (Fig 1).^2,25^ In addition to the release of chemical substances from dental materials, the biological effects of released nanoparticles (size: 1 to 100 nm) are increasingly being discussed. These are found both in the environment and in everyday products, such as sunscreen.^22,23^ On the one hand, nanoparticles are deliberately added to composites in order to improve properties such as polishability; on the other hand, they can also be generated during the milling process of larger filling particles and thus unintentionally enter the materials.^22,23^ In addition, the dentist produces nanoparticles during grinding, polishing or removal of restorations (Fig 2), even if the material initially does not contain any.^4^ For amalgam, no data are available on the formation of nanoparticles during processing with rotary instruments. Although in older studies on the removal of amalgam restorations, the nano range was not metrologically recorded according to today’s standards, the data from those studies, together with the knowledge of machining processes, leads to the conclusion that nanoparticles are also produced here.^7^ However, the mere exposure or presence of a substance in tissue (e.g. from a material) does not inevitably lead to a biological effect or even damage to health. A decisive role is played by the effective concentration, which depends on various parameters: amount of substance released per unit of time; diffusion and transport to the target tissue; detoxification and elimination kinetics.

The largest amounts of substances are released from both amalgam and composite restorations immediately after curing, but this usually decreases over time. The diffusion properties and transport of substances to the target tissue influence their effective concentration as well as their biological effect. For example, the residual dentin layer at the cavity floor represents a diffusion barrier to the dental pulp. The concentration of released substances from amalgam or composite is therefore much higher at the cavity floor than in the underlying pulp tissue.

In addition, detoxification and excretion kinetics as well as the storage behaviour of the organs involved influence the ultimate effective concentration. Of particular interest are the effects of very low concentrations, because even small amounts of substances can lead to allergic reactions in sensitised patients. Effects are also attributed to bisphenol A at low concentrations, since it can develop a hormone-like effect by binding to estrogen receptors. The EU limit for the permissable daily intake of bisphenol A was lowered in 2015 from 50 µg/kg body weight to a temporary Tolerable Daily Intake (t-TDI) of 4 µg/kg body weight.^13^ This is in line with the recommendation of the European Food Safety Authority (EFSA). A total uncertainty factor of 150 (for inter- and intra-species differences and uncertainty in mammary gland, reproductive, neurobehavioral, immune and metabolic system effects) was applied to establish a temporary Tolerable Daily Intake (t-TDI) of 4 µg/kg body weight per day.^5,14,28^ Currently, an EU commission is working on a renewed review of this limit. In December 2021, EFSA proposed a new limit for TDI that is 0.04 ng/kg body weight, about 100,000 times lower than the 2015 value.

Due to the increased and regular exposure to the released components, dental personnel as a whole must be considered a risk group.

## Oral Symptoms

**Fig 3:**
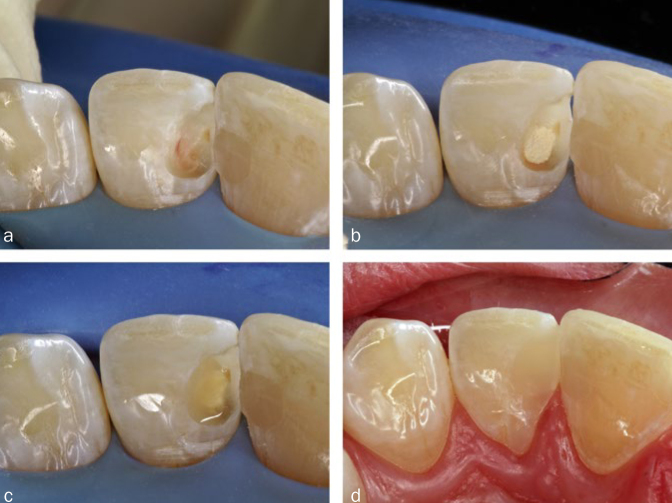
Direct pulp capping. (a) The dental pulp was exposed over a small area during caries excavation under rubber-dam isolation. Neither pulpitis symptoms prior to treatment nor pronounced bleeding were present. (b) The cavity was disinfected, and the exposed pulp covered with modified tricalcium silicate cement (Biodentine, Septodont; Niederkassel, Germany). (c) After initial setting, the bioactive cement was coated with a self-adhesive, light curable and flowable composite (Vertise Flow, Kerr; Orange, CA, USA). (d) The composite covering of the bioactive cement made it possible to continue working and to condition the cavity immediately, so that the definitive filling could be completed without delay.

**Fig 4:**
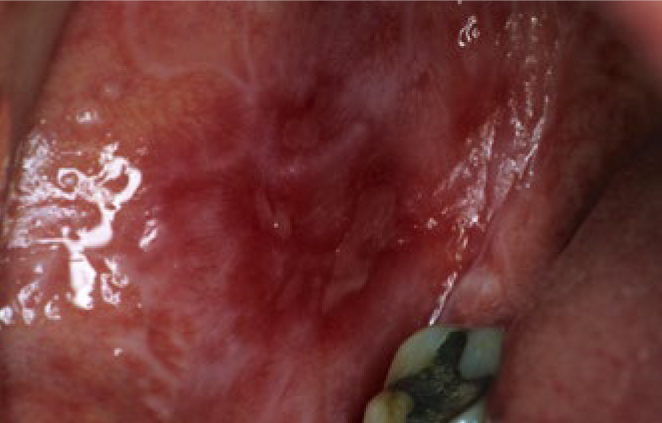
Oral lichenoid reaction at the buccal mucosa in the vicinity of an occlusal amalgam filling.

**Fig 5:**
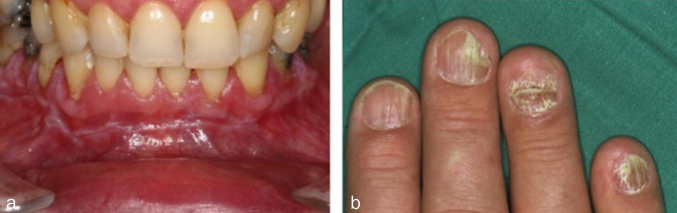
Oral lichen planus. (a) Oral lichen planus not associated with dental materials in the region of the entire mandibular vestibule. (b) Extraoral manifestation of lichen planus on the fingernails.

Postoperative hypersensitivities have been described for both amalgam and composites, although the causes are assumed to be less due to toxicity than to the high thermal diffusivity (amalgam) or to liquid displacements in the dentinal tubules caused by microgaps (composites). Irreversible pulp damage is not to be expected in the case of flat cavities or cavities far from the pulp, neither with amalgam nor with adhesively cemented composite, because dentin is a sufficient barrier. The meticulous application of the adhesive technique also prevents the penetration of bacteria into the dental pulp, thus protecting it. However, in the case of deep cavities close to the pulp, with the risk of the pulp exposure both under amalgam and composite restorations, a material should be applied that protects the pulp and stimulates tertiary dentin formation. Some examples of such bioactive materials are calcium hydroxide or tricalcium silicate cements, such as mineral trioxide aggregate (MTA) and Biodentine (Fig 3), whereas monomers released from adhesives and composites prevent the formation of tertiary dentin.^20^ Increased bacterial accumulation on composite surfaces can lead to gingivitis; in contrast, antimicrobial properties have been attributed to amalgam.^13^ Furthermore, in rare cases, both restoratives can cause oral lichenoid reactions (OLR) of the oral mucosa, which appear as localised, whitish and non-wipeable alterations (Fig 4). Oral lichen planus (OLP) should be distinguished from OLR, which often appears generalised in the oral cavity and shows reticular white stripes (Wickham stripes) (Fig 5a). Extraoral manifestations of lichen planus are seen around the fingernails (Fig 5b). In case reports, localised OLR was mostly described in contact with amalgam, more rarely with composites. The causes were mechanical irritation and allergic reactions to material components. In contrast to OLR, OLP is not associated with materials present in the mouth.^18^

## Allergies

**Fig 6:**
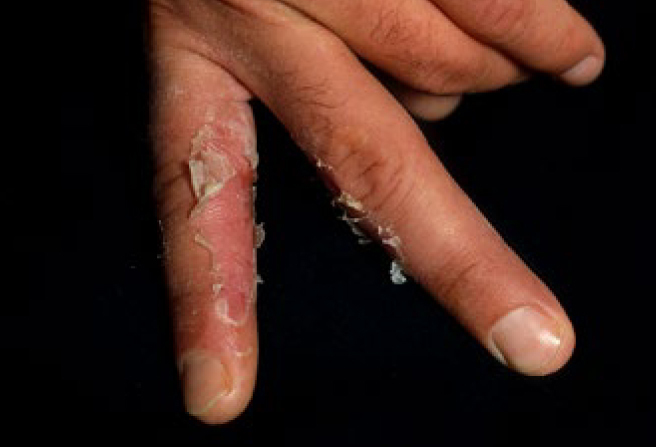
Contact allergy of a dentist to composite resins.^21^

Cases of both immediate (type 1, minutes after exposure) and delayed (type IV, days after exposure) allergic reactions have been described for both amalgam and composites.^18,21^ Allergies to acrylates such as TEG-DMA or HEMA have been observed in particular among dental personnel (ca 2%) (Fig 6). Conventional latex or vinyl gloves do not offer adequate protection, as monomers can penetrate them within a few minutes. For this reason, dental personnel are recommended to avoid any contact with composites even when wearing treatment gloves (no-touch technique). The frequency of allergic reactions in patients is considerably lower: although exact figures are lacking, a side-effect rate (mostly local) of 0.3% is currently assumed for all dental materials.^13^

## Mercury vs Bisphenol A

The mercury in amalgam was and is said to have systemic effects, ie, general poisoning; in particular, neurological diseases and kidney damage are said to be associated with it. A particular sensitivity to mercury has been described in children.^13^ Numerous national and international commissions have investigated this question, most recently in 2015, in a comprehensive analysis by an EU commission (SCENIHR). Here, too, the above-mentioned suspicions could not be substantiated for the general population (cf. section on ‘Risk groups’).^1^

**Fig 7:**
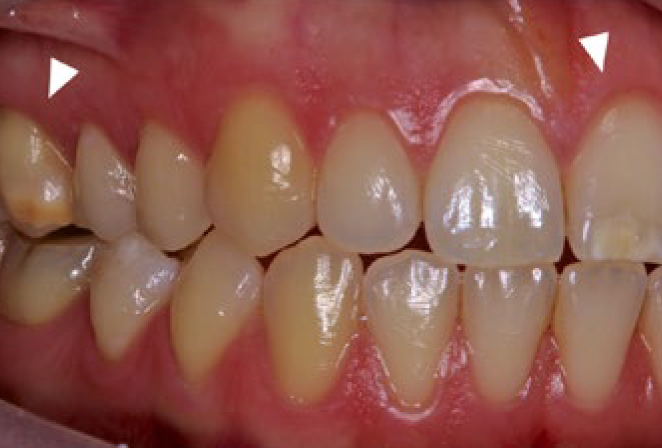
In animal studies, mineralisation disorders similar to human molar-incisor hypomineralisation (arrowheads) were found after bisphenol A administration, although the clinical relevance of these results is unclear.^26^

In analogy to mercury in amalgam, bisphenol A is released from composites and has been blamed for a number of disorders, such as reduced fertility, premature onset of puberty, diabetes and obesity.^27,29^ In the field of dentistry, the administration of bisphenol A (5 µg/kg body weight) to rats led to molar incisor hypomineralisation (Fig 7).^9,10^ However, it should be noted that the metabolism of bisphenol A in rodents differs from that in humans, in whom the lipophilic bisphenol A is bound to glucuronic acid and excreted in the urine. In rodents, on the other hand, it is secreted into the intestine, where it is reabsorbed. Thus, the effective bisphenol A concentration in rodents is fundamentally higher than in humans. SCENIHR (Scientific Committee on Emerging and Newly-Identified Health Risks) recently evaluated the tolerability of bisphenol A in medical devices, including composite materials.^14^ It was concluded that the release of bisphenol A from dental materials poses a negligible risk.^14^ In a 2014 study by the American Dental Association (ADA), it was found that the release of bisphenol A – also from materials containing bis-DMA – is orders of magnitude below the current EU oral intake limit of 4 µg/kg body weight.^1^ Removal of the superficial, non-polymerised resin layer after placement of the restoration/sealant also reduces bisphenol A exposure. However, the new TDI limit for BPA of 0.04 ng/kg body weight proposed in 2021 would necessitate a new risk assessment for resin-based restorative materials.

## Nanoparticles

Nanoparticles can lead to toxic reactions in laboratory experiments, e.g. through the formation of reactive oxygen species (ROS). Clinical symptoms after inhalation of nanoparticles have so far only been described in the field of dentistry for dusts in dental laboratories, if the recommended protective measures were not applied. Worst-case calculations of dust exposure during grinding and polishing of composite restorations have shown that the exposure of both dental staff and patients is far below the permissible occupational exposure limits and only a fraction of the usual background exposure.^22,24^ However, further data on patients with risk diseases, such as chronic obstructive pulmonary disease (COPD) or asthma, are lacking. The World Dental Federation (FDI) recently adopted recommendations to minimise dust exposure in dental practices. Table 1 lists measures that practitioners can take to reduce the generation of dusts and nanoparticles and to the minimise exposure to generated particles in order to protect the patient and dental personnel.^8,23,26^

## Risk Groups

**Fig 8:**
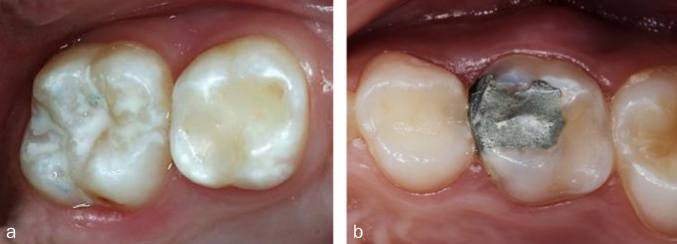
Direct restorations of deciduous teeth. (a) Restorations or fissure sealants on deciduous teeth are usually made of composite resin, resin-containing materials or glass ionomer cements (single-surface cavities).^19^ (b) Placing amalgam fillings on deciduous teeth is now only permitted in certain cases according to the EU regulation of May 2017.

When assessing biocompatibility, special risk groups can be defined in addition to the general population. These are characterised by increased exposure (e.g. dental personnel) or certain life circumstances (e.g. sensitisation, pregnancy, certain diseases), which require special precautions. Dental personnel are subject to high exposure to non-cured materials and machining dusts of hardened materials. The protective measures include the ‘no-touch technique’ mentioned above and precautions to reduce dust exposure (Table 1). Children are often regarded as a risk group because of their low body weight and possibly immature immune system. However, no particular risk has been identified for amalgam (see above). This finding is based i.a. on studies in which a large number of children received fillings made of amalgam, compomer, or composite and no clinical signs of neurological problems or renal damage were observed.^3,6^ Some studies have found evidence of discrete changes in renal markers, but their clinical relevance is controversial.^13^ Nevertheless, the EU Scientific Commission SCENIHR did not consider amalgam as a first-choice material for deciduous teeth. The reason for this assessment was the time-limited retention period of deciduous teeth in the oral cavity, which means that the higher longevity of amalgam fillings, e.g. in the case of difficult moisture control, does not play a role compared to restorations made of composite (Fig 8). On the other hand, the restriction of their use in deciduous teeth fulfilled the objective of mercury reduction specified in the Minamata Convention. The further restriction of the use of amalgam in young people under 15 years of age, as recently decreed by the EU Commission, is not included in the SCENIHR report mentioned above, and thus appears rather to be politically motivated.^13^ Pregnant and breastfeeding women are usually also considered a risk group. However, there are no data for either amalgam or composite that prove fetal harm.^13^ SCENIHR has stated that any medical or dental treatment for pregnant women should be carried out with particular caution.^13^ Therefore, extensive dental procedures should be avoided during pregnancy and conventional glass-ionomer cements should be used as a direct filling material.^19^ SCENIHR sees no reason to avoid the use of conventional filling materials during the breastfeeding period, which indicates the primarily political motivation of the relevant EU regulation.^13^ According to this regulation, amalgam must not be used in pregnant and breastfeeding women, unless the dentist considers such treatment to be absolutely necessary because of specific medical requirements of the respective patient. Patients with certain diseases also represent a risk group. For example, amalgam should not be used for persons with severe kidney disease because of the reduced excretion of mercury.^13^ However, this EU regulation allows exceptions for some medical indications, which has also been pointed out in the literature.^19^

**Table 1:** Measures for the further reduction of nanoparticles in the dental practice (adapted from Schmalz and Widbiller)^8,23,26^

Measure	Effect
Precise modeling	Reduction of the dust quantity
Sufficient water cooling	Binding of (nano)particles during the material processing
High-volume vacuum extraction	Removal of (nano)particles
Mouth-nose protection (MNP)	Exposure reduction
Capsuled materials	Reduction of dust generation during mixing
Firm connection of implant and abutment	Avoidance of titanium abrasion due to loosened implant-abutment connection
Good ventilation of the treatment areas	Reduction of nanoparticles in indoor air

## Environment

The issues of biocompatibility are directly related to environmental aspects, not least because of the Minamata Convention, which considers the ecological burden of mercury. The release of amalgam particles into the environment, e.g. during the removal of old amalgam restorations, is significantly reduced by so-called amalgam separators. In a number of European countries, such devices have been in use for a long time, with an effectiveness of over 95%. However, the processing of composites also gives rise to dusts that are directly discharged into wastewater via the high-volume extraction system and thus into the environment. A scientific commission of the EU has not yet been able to assess the extent of environmental pollution caused by such plastic particles due to a lack of data.^15^

## Emotional Risk Assessment and Risk Communication

Questions regarding the biocompatibility of amalgam and composites are primarily evaluated on the basis of clinical studies and patients are informed accordingly. In the context of the amalgam discussion, however, a fundamental discrepancy often emerged between the study results and assessments of scientific commissions on the one hand and the wishes and ideas of patients on the other. In addition, the internet offers a large body of information for patients, where both amalgam and composite resins are often subjected to unsubstantiated criticism. Patients express their reservations about amalgam and ‘plastics’. A Norwegian study even suggested that after the amalgam ban, the number of patients blaming their (mostly unspecific) complaints on composites increased significantly.^13^ For the education and briefing of the patient, it is evidently not only the data situation that is important, but also the way in which the information is conveyed. Focus must be placed on the concept of risk, because patients generally assess risks intuitively and emotionally. As a rule, this assessment does not correspond to the real risk. An example from everyday life is the increased fear of flying compared to driving a car, although fatal accidents occur significantly more often with the latter. This perceived risk must be addressed during the patient interview as part of relevant risk communication. The goal of such a conversation is therefore to shift the risk perception from an emotional level to a rational one. The patient’s fears and concerns must always be taken seriously in order to counteract the possible risk trivialisation and to maintain emotional access to the patient. Subsequently, it must be pointed out to the patient that, contrary to every illusion, life is generally not a no-risk condition. Hazards in everyday situations or during leisure activities offer the ideal opportunity to substantiate this statement (e.g. risk of accidents during sports). Finally, the risk of side effects associated with the use of dental materials (0.3% of patients) can be compared with that associated with the use of cosmetics.^13^ The latter amounts to about 12% and is thus 40 times higher than for dental materials with a similar degree of severity.^11^ In the next step, the risk should be individualised by assessing and evaluating the patient’s clinical situation. In this process, the treating dentist also puts material-related information available on the internet into the appropriate anamnestic and clinical context. In addition to medical history and local findings, this naturally includes allergies or suspected allergies, systemic diseases such as kidney disease, and pregnancy or breastfeeding. In addition, it obviously plays a decisive role that that the treating dentist knows the composition of the materials used. This is not only necessary for patients with existing or suspected allergies, but is generally important in order to be able to confidently and competently answer the patient’s questions about the composition of the materials (bisphenol A, bis-DMA). Unfortunately, the willingness of manufacturers to provide information on product components varies greatly. Legislation (e.g. the EU Medical Devices Regulation) and standardisation organisations (ISO), however, now require companies to list components comprising up to 1% and CMR components (carcinogenic, mutagenic, or toxic for reproduction) comprising up to 0.1% of the formulation.

## Conclusions

In the discussion on biocompatibility, clear parallels can be seen between amalgam and composite. Despite the restriction of indications for amalgam, the debate on the biocompatibility of dental restorative materials remains. In general, however, amalgam and composite resins can be used, taking into account the individual risk situation and the above-mentioned legal requirements. The rules of risk communication must be observed in the dialogue with the patient. Public discussion, which today is mostly focused on the materials, should be replaced by a more patient-centered approach. The individual situation of the person must be duly recognised, because individual patient factors may play an important role, e.g. allergies or existing diseases, such as impaired renal clearance. Furthermore, the subjective wishes and possible fears of the individual patient may be regarded as one such individual factor and should be addressed. Therefore, when applying the material for a single patient, all available information from anamnesis and findings, but also the patients’ own inquiries (for instance, based on their internet-acquired information) must be placed into the clinical and scientific context, which is ultimately the dentists’ task and responsibility.
